# An enhanced educational intervention for improving confidence in the eye health benefits of appropriate care for age-related macular degeneration: a randomized controlled trial

**DOI:** 10.1093/her/cyaf029

**Published:** 2025-07-04

**Authors:** Elisa Wang, Gordon S Doig, Angelica Ly

**Affiliations:** Centre for Eye Health, Rupert Myers Building, Gate 14 Barker Street, University of New South Wales, Sydney, NSW 2052, Australia; School of Optometry and Vision Science, Rupert Myers Building, Gate 14 Barket Street, University of New South Wales, Sydney, NSW 2052, Australia; Northern Clinical School, Royal North Shore Hospital Intensive Care Unit, University of Sydney, St Leonards, NSW 2065, Australia; Centre for Eye Health, Rupert Myers Building, Gate 14 Barker Street, University of New South Wales, Sydney, NSW 2052, Australia; School of Optometry and Vision Science, Rupert Myers Building, Gate 14 Barket Street, University of New South Wales, Sydney, NSW 2052, Australia

## Abstract

Age-related macular degeneration (AMD) is a leading cause of irreversible vision loss worldwide. Appropriate care is available for patients, reducing the risk of AMD progression. Unfortunately, patients do not always receive appropriate eye care. Our study aimed to develop and evaluate an enhanced educational intervention focused on the health benefits expected from receiving appropriate eye care for AMD. We conducted a randomized, single-blind, controlled trial between May 2022 and October 2023 at an intermediate-tier not-for-profit clinic, the Centre for Eye Health. We recruited 137 patients previously diagnosed with intermediate or advanced (neovascular, geographic atrophy) AMD. Patients were enrolled and randomized (68 enhanced education, 69 standard care). On the intention-to-treat analysis, there was no significant difference between groups with regards to the primary outcome, *confidence* in the eye health benefits of AMD-related care at 6 months (*P* = .25). On *a priori*-defined subgroup analysis, enhanced education resulted in a clinically meaningful and statistically significant differential improvement in *confidence* in the eye health benefits of AMD-related care for patients who were diagnosed with AMD less than 5 years ago (*P*_interaction_ = .036). Further study is needed to confirm whether enhanced education can improve *confidence* in eye health care benefits for newly diagnosed AMD patients. Trial registration: anzctr.org.au Identifier: ACTRN12622000984796.

## Introduction

Age-related macular degeneration (AMD) is a major cause of vision loss worldwide [1]. The number of people affected by AMD is projected to increase from 196 million (in 2020) to 288 million (in 2040) [2]. There are almost 2 million people of legal blindness worldwide caused by AMD, with approximately 8.4 million people with moderate or severe vision impairment [3]. One in seven people over the age of 50 in Australia show some evidence of AMD [1].

To reduce the risk of AMD progression, appropriate eye care for AMD can include nutritional supplements [4], anti-vascular endothelial growth factor (anti-VEGF) injections [5, 6], and smoking cessation [7]. The 2001 Age-Related Eye Disease Study documents that nutritional supplementation can reduce the relative risk of progression by 25% for patients with intermediate AMD (in one or both eyes) and unilateral advanced (geographic atrophy) AMD [8]. Large-scale randomized controlled trials have shown anti-VEGF injections are effective and safe in treating neovascular AMD and preserving vision [9–12], and longitudinal cohort studies document that continued smoking is associated with an increased risk (odds ratio: 2.4 to 4.8) of progression to late AMD [7, 13–15]. Unfortunately, patients with AMD do not always receive appropriate usual eye care.

Studies report up to 62% of patients with intermediate AMD do not take nutritional supplements [16] whilst 37% of patients with advanced (neovascular) AMD are non-adherent to their anti-VEGF injection schedules [17]. Many patients claim their clinicians never recommended key aspects of care appropriate to their stage of AMD [18] whilst other patients simply ‘do not recall’ being given advice for care by their clinician [19]. Even when patients do remember being given care advice, they cannot recall specific health benefits arising from the care they were recommended to receive [20] or they have unrealistic expectations of the benefits of care [21].

The aim of this study was therefore to develop an enhanced educational intervention that specifically focused on the health benefits expected to arise from appropriate AMD-related eye care. We conducted a randomized controlled trial to compare the effects of this enhanced educational intervention to standard care. The primary outcome of this study was *confidence* in the eye health benefits of AMD-related care.

## Methods

Centre for Eye Health (CFEH) patients were eligible for the study (trial registration: anzctr.org.au ACTRN12622000984796) if they already had a diagnosis of intermediate or advanced AMD recorded in their medical records by a CFEH clinician, and they had an impending routine follow-up visit for monitoring AMD progression to be scheduled at CFEH.

CFEH is a not-for-profit service provided by Guide Dogs and the University of New South Wales at no cost to patients in New South Wales [22]. CFEH is a referral-only, intermediate-tier clinic that provides advanced diagnostic imaging and management of chronic ocular conditions (e.g. glaucoma, diabetic retinopathy, AMD, etc.) [22].

For the purposes of this study, intermediate AMD was defined as a diagnosis of AMD recorded in CFEH clinical records with (i) large drusen reported at the macula in either eye and/or; (ii) pigmentary abnormalities associated with at least medium-sized drusen reported at the macula in either eye. Advanced AMD was defined as the recorded presence of geographic atrophy and/or macular neovascularization in a patient meeting the above diagnosis of AMD.

Adults over 49 years old who could communicate effectively in English and were considered legally capable to provide written informed consent were approached for participation. Patients with a co-morbid ocular disease requiring treatment (e.g. glaucoma, diabetic retinopathy) or who were intending to move outside of the study catchment area (New South Wales, Australia) over the duration of the study were excluded. Ethical approval was obtained from the University of New South Wales Human Research Ethics Committee (HC 210745 October 2021). Direct written informed consent was documented in accordance with local and international laws.

Allocation concealment was maintained by the use of sequentially numbered opaque sealed envelopes [23]. Simple one-to-one randomization [23] was used to generate the randomization sequence and to ensure overall group sizes were similar [24].

### Enhanced education

All study intervention participants were seen during a routine follow-up visit for monitoring the progression of previously diagnosed AMD. In addition to their usual optometric care, study intervention patients received enhanced education during routine follow-up clinical assessment. The main elements of the enhanced education intervention included: exposure to colourful posters in the examination room that emphasized aspects of care for each different stage of AMD; clearly worded take-home evidence summary documents (trifold brochures) describing care appropriate to their specific stage of AMD; and focused verbal education aligned with the key messages on the posters and brochures. The information included in our study’s brochures and posters used rational-based and emotional-based reasoning [25], simple wording with active sentence structure [26], and contrasting colours with red and yellow highlighting to selectively emphasize words [27]. Additionally, each poster featured one firmly worded call to action, with a verb at the front of the sentence, in line with previous research affirming the rhetorical benefits of this grammatical structure [26, 28]. For example, the main message used on the poster about nutritional supplements was: ‘Save your vision. If you have intermediate AMD, take your eye supplements every day’. Brochures and posters are accepted to be effective tools for improving patients’ health-related knowledge [29, 30]. Providing patients with verbal education and educational materials, rather than verbal information only, is known to be more effective in increasing patients’ health-related knowledge [31]. The focused verbal education delivered in our educational intervention used principles such as the primacy effect [32], provided important information first [33], ordered information with ‘bad news’ shared before ‘good’ news [34], tailored information relevant to patients’ disease stage [35], and repeated its messaging [36]. The verbal education was also designed to build rapport with patients by specifically creating pauses for the patient to ask questions [37].

Study intervention patients also received: a take-home bottle of nutritional supplements (if applicable); reminder text messages at 3 months and 5 months after their baseline visit reinforcing care relevant to the patient’s specific stage of AMD; and access to an AMD resources website developed by the authors (https://EyeHealth.EvidenceBased.net). The authors developed the educational content used during the focused verbal education and the educational content and style of the study posters, brochures, and text messages. An example of a text message sent to patients requiring anti-VEGF injections was: ‘Hi [insert patient’s name], eye injections stop you from going blind. If you start getting eye injections, do NOT stop. The Centre for Eye Health cares about you’. The AMD resources website (https://EyeHealth.EvidenceBased.net) provided links to additional information and resources developed by credible bodies such as New South Wales Health, Guide Dogs, the Macular Disease Foundation of Australia, and others. The enhanced educational intervention was delivered by the first author (E.W.) who is a registered optometrist undertaking graduate training at CFEH.

### Standard care

All standard care participants were seen during a routine follow-up visit for monitoring the progression of previously diagnosed AMD. In addition to their usual optometric care, standard care patients received pragmatic standard verbal education as determined appropriate by the attending clinician. They were not exposed to any study posters or brochures and did not receive text message reminders or access to the AMD resources website developed by the study investigators. Standard care was delivered by the first author (E.W.) who is a registered optometrist undertaking graduate training at CFEH.

### Outcomes

The primary outcome was a composite measure of *confidence* in the eye health benefits of AMD-related care, compared between groups (enhanced education and standard care) at 6 months after study enrolment. The composite measure was composed of the sum of individual care elements measuring *confidence* in the eye health benefits of smoking cessation (for smokers), use of an Amsler grid, use of nutritional supplements, and receiving anti-VEGF eye injections. *Confidence* in each individual care element was measured using a balanced 5-point Likert scale question.

The secondary outcomes included each individual care element of the composite primary outcome: *confidence* in the eye health benefits of smoking cessation, *confidence* in the eye health benefits of using an Amsler grid, *confidence* in the eye health benefits of taking nutritional supplements, and *confidence* in the eye health benefits of receiving anti-VEGF eye injections. All outcomes were compared between groups at 6 months after enrolment.

Tertiary outcomes included other measures of the patient’s self-rated general health, self-rated eye health, and self-rated assessments of their ability to find, understand, judge, and use AMD-related information.

At the study baseline, a questionnaire was given to patients prior to receiving any intervention and eye care. The selection and wording of individual questions were adapted from components of validated instruments, including the Australian Census Household Form [38], the RAND 36-Item Short-Form Health Survey [39, 40], Macular Disease Society Questionnaire [41], the European Health Literacy Survey Questionnaire HLS-EU-Q47 [42], and the Health Information National Trends Survey [43]. A pilot test of a draft questionnaire was performed with 12 participants with AMD and 12 participants with other chronic eye diseases (i.e. glaucoma, etc.) to improve readability [44]. Feedback was also provided by two CFEH researchers with expertise in AMD who were not involved in the conduct of the study.

Enrolled patients were followed up 6 months post-randomization unless consent was withdrawn. At the 6 month follow-up appointment, patients were given a follow-up questionnaire with the same questions as the baseline questionnaire before proceeding to their usual clinical assessment. There were no financial incentives for participating in the study.

### Sample size, power, and statistical analysis

Assuming an SD of 1 for the primary outcome *confidence* in the eye health benefits of AMD-related care [45], it was calculated that 126 patients (63 patients in each group) would provide greater than 90% power to detect a moderate sized treatment difference between groups of a 0.5 [46, 47]. The initial estimate of 126 patients was increased to 130 patients to protect against loss of power due to unavoidable drop-outs (e.g. withdrawal of consent, deaths, etc.).

A detailed intention-to-treat analysis plan has been published elsewhere [48]. All analyses were conducted under the principle of intention to treat, with all randomized patients analysed in their allocated groups. The primary outcome was analysed using linear regression to compare the difference in *confidence* in the eye health benefits of AMD-related care between the two groups, collected at 6 months after enrolment, whilst controlling for baseline *confidence* in the eye health benefits of AMD-related care as a covariate [49, 50]. The magnitude of the treatment effect was calculated as the absolute difference between groups, with 95% confidence intervals (CI). In secondary analyses, a pre-specified algorithm was used to identify baseline variables for inclusion in a covariate-adjusted regression model [51].

Missing individual components of the composite primary outcome were accepted to be missing at random and not included in the primary analysis [52] unless greater than 5% of all individual components for analysis were found to be missing. In the case of excessive missing outcomes, missing values were imputed using study group-specific pooled mean values [48].

Thresholds for a minimal clinically important difference for the primary outcome and other health-related quality-of-life measures were defined *a priori* [48]. Using the approach proposed by Juniper *et al.* [53] and validated by Norman *et al.* [54], a minimal clinically important difference was defined as one-half of the SD of the pooled results from that measure [55]. Differences in the magnitude of the effect that were greater than 1 SD were described as having a *moderate* effect, and differences greater than 1.5 SDs were described as having a *large* effect [53].

Four *a priori* subgroup analyses described in the Statistical Analysis Plan, which was published before the study close out, were conducted based on (i) median duration of AMD, (ii) stage of AMD, (iii) median score for self-reported quality of eyesight, and (iv) median score for self-reported health literacy [48].

Two-sided 5% significance levels were used to identify statistically significant results. All statistical analyses were conducted in SPSS (version 26; IBM).

## Results

**Figure 1:**
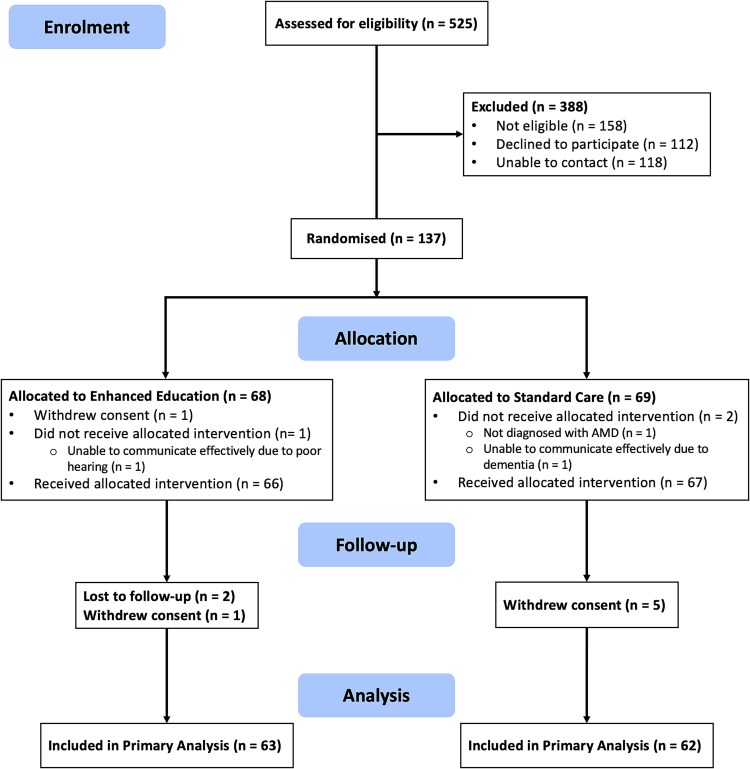
Patient recruitment flow diagram.

From 1 May 2022 to 26 October 2023, 525 patients were assessed for eligibility to participate in the study. Of these 525 potentially eligible patients, 158 were determined to be not eligible for participation, 118 could not be contacted, and 112 declined to participate. Of the 137 eligible patients who consented to enrolment, 125 successfully completed the trial and were included in the primary analysis [48]. Figure 1 presents complete details of patient flow throughout all stages of the trial.

The mean (SD) age of enrolled patients was 74.9 (7.1) years and 62% (78/125) were females. Stage of AMD was 90% (112/125) intermediate AMD, 8% (10/125) advanced AMD with geographic atrophy, and 2% (3/125) advanced neovascular AMD. Table 1 presents patient baseline characteristics by study group.

**Table 1:** Patient characteristics and baseline balance.

**Baseline variables**	**Enhanced education (*N* = 63)**	**Standard care (*N* = 62)**	***P*-value**
**Age,** in years, mean (SD)	73.6 (7.46)	76.1 (6.53)	.06
**Gender,** female, % (*n*/*N*)	66.7 (42/63)	58.1 (36/62)	.32
**Education level**			
College degree or higher, % (*n*/*N*)	52.4 (33/63)	48.4 (30/62)	.66
**Stage of AMD,** worse eye, % (*n*/*N*)			
Intermediate	88.9 (56/63)	90.3 (56/62)	Referent
Advanced (GA)	7.9 (5/63)	8.1 (5/62)	.86
Advanced (neovascular)	3.2 (2/63)	1.6 (1/62)	
**Duration of AMD**, in years, mean (SD)	5.00 (4.70)	6.44 (5.51)	.07
**Current smoker**, % (*n*/*N*)	6.3 (4/63)	8.1 (5/62)	.74
**Chronic medical condition**, % (*n*/*N*)			
Cardiovascular disease	25.4 (16/63)	21.0 (13/62)	.56
Cancer	19.0 (12/63)	22.6 (14/62)	.63
Arthritis	42.9 (27/63)	48.4 (30/62)	.54
Cataracts	11.1 (7/63)	8.1 (5/62)	.56
**SF-36 general health self-rating**, mean (SD)	3.46 (0.82)	3.50 (0.82)	.79
**General eyesight self-rating**, mean (SD)	2.87 (0.83)	2.81 (0.72)	.63
**General eyesight self-rating compared with 6 months ago**, mean (SD)	2.81 (0.47)	2.95 (0.66)	.17
**General Health Literacy Index**, mean (SD)	3.36 (0.67)	3.51 (0.67)	.23

Abbreviations: *N*, number of participants; SD, standard deviation; AMD, age-related macular degeneration; GA, geographic atrophy; SF-36, 36-Item Short Form Health Survey.

### Primary outcome

On primary analysis controlling for study baseline *confidence* in the eye health benefits of AMD-related care, there was no difference between the enhanced educational intervention and standard care groups with regards to the primary outcome, *confidence* in the eye health benefits of AMD-related care at 6 months (0.10, 95% CI: −0.07 to 0.27, *P* = .25, Table 2).

**Table 2:** Primary and secondary outcomes at 6-month follow-up.

**Variable**	**Enhanced education (*N* = 63)**	**Standard care (*N* = 62)**	**Difference (95% CI)**	***P*-value**
**Primary outcome**				
*Confidence* in the eye health benefits of AMD-related care, mean (SD)	4.26 (0.49)	4.16 (0.59)	0.10 (−0.07 to 0.27)	.25
**Secondary outcomes**				
*Confidence* in the eye health benefits of quitting smoking, mean (SD)	4.47 (0.62)	4.35 (0.73)	0.12 (−0.13 to 0.37)	.35
*Confidence* in the eye health benefits of taking nutritional supplements for intermediate AMD, mean (SD)	4.40 (0.55)	4.24 (0.67)	0.16 (−0.06 to 0.38)	.16
*Confidence* in the eye health benefits of using an Amsler grid regularly, mean (SD)	4.17 (0.64)	4.11 (0.68)	0.06 (−0.16 to 0.28)	.60
*Confidence* in the eye health benefits of ongoing eye injection treatments for wet AMD, mean (SD)	4.00 (0.81)	3.94 (0.79)	0.06 (−0.19 to 0.31)	.65

Abbreviations: *N*, number of participants; SD, standard deviation; AMD, age-related macular degeneration.

Covariate-adjusted analysis of the primary outcome controlled for baseline age, self-reported general eyesight, self-reported health literacy, and *confidence* in the eye health benefits of AMD-related care failed to find any difference between groups with regards to 6-month *confidence* in the eye health benefits of AMD-related care (0.09, 95% CI: −0.07 to 0.25, *P* = 0.25). There were 34 instances where imputations were performed using pooled mean values due to missing values. The maximum number of missing values for any one variable was 7.2% (9/125).

### Secondary outcomes

As reported in Table 2, at 6 months, there were no significant differences between groups with regards to the patient’s *confidence* in the eye health benefits of quitting smoking (*P* = .35), taking nutritional supplements (*P* = .16), using an Amsler grid (*P* = .60) or receiving anti-VEGF eye injections (*P* = .65).

### Tertiary outcomes

There were no differences between groups at 6 months with regards to any tertiary outcome. See Table 3 for complete details.

**Table 3:** Tertiary outcomes at 6-month follow-up.

**Tertiary outcome variables, mean (SD)**	**Enhanced education (*N* = 63)**	**Standard care (*N* = 62)**	**Difference (95% CI)**	***P*-value**
SF-36 general health self-rating	3.35 (0.72)	3.39 (0.75)	−0.04 (−0.31 to 0.23)	.78
General eyesight self-rating	2.84 (0.68)	2.79 (0.73)	0.05 (−0.19 to 0.12)	.69
General eyesight self-rating compared with 6 months ago	2.86 (0.35)	2.87 (0.46)	−0.01 (−0.10 to 0.09)	.85
General Health Literacy Index	3.63 (0.68)	3.72 (0.70)	−0.09 (−0.33 to 0.15)	.48
Ease of *finding* general AMD-related information	3.71 (0.70)	3.78 (0.75)	−0.07 (−0.31 to 0.17)	.59
Ease of *understanding* general AMD-related information	3.86 (0.70)	3.86 (0.64)	0.00 (−3.5 to 3.5)	.98
Ease of *judging* general AMD-related information	3.43 (0.66)	3.48 (0.66)	−0.05 (−0.29 to 0.19)	.69
Ease of *using* general AMD-related information	3.68 (0.67)	3.67 (0.76)	0.01 (−0.22 to 0.25)	.94
*Trust* in general information about macular degeneration from an optometrist	4.19 (0.59)	4.18 (0.70)	0.01 (−0.19 to 0.21)	.93

Abbreviations: SD, standard deviation; *N*, number of participants; SF-36, 36-Item Short Form Health Survey; AMD, age-related macular degeneration.

### Subgroup analyses

Four *a priori* planned subgroup analyses were undertaken (described in the Statistical Analysis Plan [48], which was published before the study close out). Based on a formal test of interaction, there was a significant differential treatment effect based on the duration of AMD: enhanced education resulted in a clinically meaningful and statistically significant differential improvement in confidence in the eye health benefits of AMD-related care for patients who were diagnosed with AMD less than 5 years ago (0.399, 95% CI: 0.027 to 0.770, *P*_interaction_ = .036). There were no significant differential treatment effects based on stage of AMD (*P*_interaction_ = .408), self-reported quality of eyesight (*P*_interaction_ = .701) or self-reported health literacy (*P*_interaction_ = .412).

## Discussion

This randomized controlled trial allocated patients with AMD to receive either an enhanced educational intervention or standard care. The enhanced educational intervention specifically focused on the health benefits expected to arise from appropriate AMD-related eye care. The main elements of the enhanced educational intervention included colourful exam room posters, clearly worded take-home brochures, and focused verbal education aligned with the key messages on the posters and brochures.

On the intention-to-treat analysis, the enhanced educational intervention did not result in significant improvements to patients’ *confidence* in the eye health benefits of AMD-related care. However, on *a priori*-defined subgroup analysis, enhanced education resulted in a clinically meaningful and statistically significant differential improvement in *confidence* in the eye health benefits for patients who were diagnosed with AMD less than 5 years ago (0.399, 95% CI: 0.027 to 0.770, *P*_interaction_ = .036).

### Education and time of diagnosis

This study’s finding of a clinically meaningful differential effect of enhanced education on newly diagnosed patients is in line with previous research. A 2008 study by Tien *et al.* evaluated the impact of continuing diabetic education on the HbA1c levels of 211 patients. They found that only patients with a shorter duration of diabetes at the time of education were able to maintain significantly better glycaemic control over the 1-year study follow-up period [56]. The authors concluded their study by recommending that diabetic education should be offered to patients as close to the time of diagnosis as possible [56]. It is now standard practice for education for patients with diabetes [57] and many other chronic diseases [58, 59] to receive education as close to the time of diagnosis as possible. Our subgroup analysis, demonstrating the differential effect of enhanced education on patients with a shorter duration of AMD, was planned before the study close out [48] and is mechanistically plausible [60].

The information needs of patients are accepted to change over the course of their disease [61] and are also altered by treatment-related events [62]. Our educational strategy focused on issues related to disease progression and the benefits of starting treatment. It did not focus on complications arising from ongoing treatment. It is plausible that our educational strategy had a minimal effect on patients with a longer duration of disease because it did not address the treatment-related information needs of patients already receiving care [63]. More research is needed to better understand how information needs change over time for patients with AMD.

### Changing attitudes leads to changing behaviours

According to the Transtheoretical Model, an individual’s health behaviour will change only after they understand and accept the health benefits of changing [64, 65]. The antecedent requirement for a belief in health benefits is widely accepted in the field of smoking cessation [66]. Indeed, multiple studies show that increased exposure to information that primarily focuses on the physical benefits of quitting smoking is strongly associated with increased attempts to quit and increased successful quitting [67–69]. Under the tenets of the Transtheoretical Model, improving a patient’s *confidence* in the benefits of AMD-related care takes the patient one step closer to changing their health behaviour by seeking that desirable care. Future evaluations of enhanced educational interventions should investigate whether this change in attitude does translate into a change in care-seeking behaviour for patients with AMD.

### Strengths and limitations

This educational randomized controlled trial was designed, executed, and reported in compliance with major guidance standards for medical intervention trials: a detailed protocol for statistical analysis was published before the study close out [48, 70], and execution and reporting were guided by the CONSORT 2010 statement [71]. Application of key guidance documents led to the use of blinding at the time of analysis, helped minimize loss to follow-up, and prevented treatment cross-overs.

It is important to note that the same person delivered the enhanced educational intervention and provided education to the standard care group (first author, E.W.). This may have resulted in study intervention and standard care groups receiving similar verbal education, even though the management committee guided E.W. to remove ‘key phrases’ used during the verbal component of the enhanced education intervention from her standard care verbal education. The use of the same person to deliver verbal education to both study groups may not be a weakness. Similar intensities of verbal education in each group highlight the remaining important differences: patients allocated to standard care did not receive brochures or text messages and did not see any study posters.

Another limitation of this study was the uneven group sizes for the different stages of AMD. Most patients in this trial had intermediate AMD, which further impacts the already small overall sample size, almost ensuring non-significant results in other key subgroups of patients. Additional research may be required to draw strong inferences about patients with a specific stage of AMD. Similarly, very few smokers (4/63) received an educational intervention on smoking cessation, so additional research may be required to draw strong inferences about this specific group of patients. Whilst it may similarly be argued that few patients receiving anti-VEGF treatment (2/63) underwent our educational intervention, we suggest that information on the importance of future anti-VEGF injections is relevant to all patients with AMD. Furthermore, the benefits of advising patients about taking nutritional supplements and anti-VEGF injections may be outweighed by the limitations of cost; however, our study did not address issues of costs. We strongly recommend future research consider and address this issue.

The CFEH is a referral-only, intermediate-tier, not-for-profit clinic that provides advanced diagnostic imaging and management of chronic ocular conditions. It is possible the patient population attending CFEH for care is unique. The education previously provided to these unique patients, who had been referred to advanced care, may also have been unique. Previous unique education may have influenced the standard care patients’ healthcare knowledge and experience, further contributing to non-significant results in this small and relatively underpowered clinical trial. Conscientious, explicit, and judicious decision-making should be used when generalizing our results to different care settings and patients with different ocular conditions.

### Future research directions

Further research is required to understand the information needs of patients with different stages and durations of AMD. Intervention studies should develop and evaluate novel educational strategies based on these information needs. Eventually, future studies should investigate cohorts of patients with AMD to understand whether AMD patients’ attitudes towards care influence their future care-seeking behaviours. Additional research is needed to understand the information needs of patients with AMD for longer than 5 years.

## Conclusion

In this rigorously conducted randomized controlled trial, we learned that enhanced education resulted in a clinically meaningful and statistically significant differential improvement in *confidence* in the eye health benefits of AMD-related care for patients who were diagnosed with AMD less than 5 years ago. Further study is needed to confirm whether enhanced education can improve *confidence* in the eye health benefits of AMD-related care for newly diagnosed AMD patients and to understand the information needs of patients with different stages of AMD.
